# Childhood attention‐deficit hyperactivity disorder: socioeconomic inequalities in symptoms, impact, diagnosis and medication

**DOI:** 10.1111/camh.12707

**Published:** 2024-03-18

**Authors:** Anna Pearce, Paul Henery, S. Vittal Katikireddi, Ruth Dundas, Alastair H. Leyland, Dasha Nicholls, Russell M. Viner, Lynda Fenton, Steven Hope

**Affiliations:** ^1^ University of Glasgow Glasgow UK; ^2^ Public Health Scotland Edinburgh and Glasgow UK; ^3^ Imperial College London London UK; ^4^ University College London London UK

**Keywords:** Social class, ADHD, lifecourse, large data

## Abstract

**Background:**

Children from disadvantaged backgrounds are at greater risk of attention‐deficit hyperactivity disorder (ADHD)‐related symptoms, being diagnosed with ADHD, and being prescribed ADHD medications. We aimed to examine how inequalities manifest across the ‘patient journey’, from perceptions of impacts of ADHD symptoms on daily life, to the propensity to seek and receive a diagnosis and treatment.

**Methods:**

We investigated four ‘stages’: (1) symptoms, (2) caregiver perception of impact, (3) diagnosis and (4) medication, in two data sets: UK Millennium Cohort Study (MCS, analytic *n* ~ 9,000), with relevant (parent‐reported) information on all four stages (until 14 years); and a population‐wide ‘administrative cohort’, which includes symptoms (child health checks) and prescriptions (dispensing records), born in Scotland, 2010–2012 (analytic *n* ~ 100,000), until ~6 years. We described inequalities according to maternal occupational status, with percentages and relative indices of inequality (RII).

**Results:**

The prevalence of ADHD symptoms and medication receipt was considerably higher in the least compared to the most advantaged children in the administrative cohort (RIIs of 5.9 [5.5–6.4] and 8.1 [4.2–15.6]) and the MCS (3.08 [2.68–3.55], 3.75 [2.21–6.36]). MCS analyses highlighted complexities between these two stages, however, those from least advantaged backgrounds, with ADHD symptoms, were the least likely to perceive impacts on daily life (15.7% vs. average 19.5%) and to progress from diagnosis to medication (44.1% vs. average 72.5%).

**Conclusions:**

Despite large inequalities in ADHD symptoms and medication, parents from the least advantaged backgrounds were less likely to report impacts of ADHD symptoms on daily life, and their children were less likely to have received medication postdiagnosis, highlighting how patient journeys differed according to socioeconomic circumstances.

**Key Practioner Messages**

**What is known?**

- Children from less advantaged backgrounds are more likely to experience attention‐deficit hyperactivity disorder (ADHD)‐related symptoms, to be diagnosed with ADHD and to be prescribed ADHD medications.

**What is new?**

- Less is known about how inequalities occur across the ‘patient journey’ and therefore where intervention points may be to ensure all families are receiving the appropriate support.
- Using a Scottish administrative cohort and the UK Millennium Cohort Study, we aimed to examine how inequalities manifest across the patient journey, from perceptions of impacts of ADHD symptoms on daily life, to the propensity to seek and receive a diagnosis and treatment.

**What is significant for clinical practice?**

- Despite there being large inequalities in ADHD symptoms and medication, parents from the least advantaged backgrounds whose children had ADHD symptoms were less likely to report impacts of those symptoms on daily life, and their children were less likely to progress from diagnosis to medication.
- The results require replication elsewhere but point towards possible points in the system where professionals may be able to intervene to ensure that all children are receiving sufficient and appropriate support.

## Background

Attention‐deficit hyperactivity disorder (ADHD) is a condition characterised by developmentally inappropriate levels of hyperactivity, impulsivity and inattention. Worldwide prevalence of ADHD is estimated at around 5%, with limited variation across Europe and North America (Polanczyk, de Lima, Horta, Biederman, & Rohde, 2007). It is relatively common in the UK for children with ADHD to have not been diagnosed (Taylor, 2017). The wider mental health literature indicates that ethnic minority families, those with lower levels of education and those living in poverty, have higher levels of unmet mental health needs (Bansal et al., 2014; Olsson, Hensing, Burström, & Löve, 2021). If ADHD goes unmanaged, it can reduce academic achievement, limit employment opportunities and possibly lead to personality disorders, substance misuse and impaired social adjustment (Wright et al., 2015). Therefore, it is important for children who experience ADHD symptoms which impact their day‐to‐day lives to receive specialist advice and support. This may include treatment interventions including, where appropriate, pharmacological treatment.

**Figure 1:**
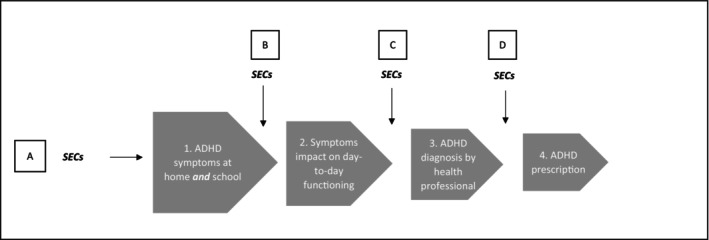
Conceptual diagram of a proposed ‘patient journey’ through four measurable ADHD stages in childhood, from symptoms to medication prescribing, with the influence of socioeconomic circumstances (SECs) at points A to D

Children from less advantaged backgrounds have consistently poorer outcomes than their more advantaged peers across a range of physical and mental health conditions (Pearce, Dundas, Whitehead, & Taylor‐Robinson, 2019), including socioemotional development and generalised anxiety and depression disorders. ADHD is no exception, with higher rates observed in less advantaged groups (Fleming et al., 2017; Russell, Ford, Williams, & Russell, 2016; Spencer et al., 2022). ADHD offers an opportunity to understand how health inequalities in childhood can manifest, since it is likely to be affected by a number of social determinants, including child and family level risk factors, public attitudes to ADHD and the capacity and quality of health systems. Despite this, few studies have formally considered how inequalities manifest between the onset of symptoms and the point of treatment. Figure 1 proposes a simplified journey that children and their families progress through. There are four, measurable ‘stages’ (presented in grey blocks): ADHD symptoms, impact, diagnosis and treatment. Points at which inequalities may be introduced are shown with small hollow boxes (A–D). The journey starts with the onset of ADHD symptoms (stage 1). ADHD symptoms are socially distributed, with children from less advantaged socioeconomic circumstances (SECs) more likely to display externalising behavioural problems, including hyperactivity (Reiss, 2013; Vaezghasemi et al., 2023). Thus, socioeconomic inequalities are initially introduced at point A. It is worth noting that there is potential for inequalities to be underestimated at this stage if recognition of symptoms is also by affected by family characteristics (Eiraldi, Mazzuca, Clarke, & Power, 2006); however, this is closely linked to perceived impact, considered in the next stage.

In most countries, it is recommended that diagnosis in children and adolescents is contingent upon ADHD symptoms impairing psychological, social, educational or occupational functioning, across multiple settings (e.g. school and home) (National Institute for Health and Care Excellence, 2017). This transition from symptoms to impact marks stage 2. Screening tools are used to identify symptoms and impacts on the child, alongside consideration of coexisting conditions and the social circumstances of the family, including the mental health of parents/carers (National Institute for Health and Care Excellence, 2017). Inequalities may be introduced at point B for several reasons. Parental perceptions of what is normative rely on interactions with other families, the views of teachers and cultures, beliefs and expectations about children's behaviour (Reardon, Harvey, & Creswell, 2020; Wright et al., 2015). This stage therefore bears similarities to the ‘problem recognition’ stage of traditional models of help‐seeking behaviours, which has been used to examine ethnic differences in ADHD services in the United States (Eiraldi et al., 2006). However, the ‘impact’ stage in our model also proposes that the true impact of ADHD‐like symptoms on a family's and child's day‐to‐day functioning depends on their socioeconomic resources, which can have consequences for parental mental health (McGinnis, Copeland, Shanahan, & Egger, 2022), family structure, social support, parenting style and childcare (Pearce et al., 2019).

Among families for whom a child's ADHD symptoms are impairing day‐to‐day functioning of the child and/or family, the decision to seek a diagnosis (stage 3) will be influenced by whether families perceive themselves to be legitimate ‘candidates’ (Mackenzie, Conway, Hastings, Munro, & O'Donnell, 2013) for support. Overdiagnosis may also occur if there have been cultural shifts in what is considered typical age‐appropriate behaviours (Kazda et al., 2021). Parents from more advantaged backgrounds may be more likely to pursue an ADHD diagnosis due to perceived benefits (from resultant medication or in‐school supports) to academic performance (Owens, 2021). Access to health services is also unequal across society (Eiraldi et al., 2006; Wright et al., 2015), due to variations in the availability of services and the size of waiting lists as well as the capacity of families to navigate those services and persist to the point of diagnosis (Eiraldi et al., 2006). This applies in settings where there is no universal healthcare, but also in countries like the UK where advantaged families are more likely to use private health care due to there being greater choice and fewer delays compared to the National Health Service (NHS). In addition, some families may be more reluctant to seek help because they have lower expectations of the support available for children with ADHD symptoms, have low trust in health services or are more likely to consider an ADHD diagnosis stigmatising (Eiraldi et al., 2006).

Finally, inequalities may be introduced at the point of treatment (stage 4). In line with the Inverse Care Law (Hart, 1971), children with ADHD symptoms from less advantaged families may be less likely to receive treatment or the most appropriate treatment for their needs (Taylor, 2017). Treatment options for ADHD include behavioural parenting interventions, child psychological therapy and stimulant and nonstimulant drugs. UK guidance stipulates that nonpharmacological treatments, such as parent‐training programmes, should be tried before progressing to medication. However, the availability and suitability of nonpharmacological treatments may vary. Less advantaged families may not fully benefit from nonpharmacological treatments due to time scarcity (Strazdins et al., 2011), inflexible travel or childcare options and potential stigma associated with the requirement for parenting training. Less advantaged families may experience higher ‘treatment burden’ if the child or other family members are also being treated for other health conditions (May et al., 2014), and they may find it harder to deal with issues that can be identified during the treatment process, such as parenting style and confidence and the underlying influences of these (which may stem from their own childhoods).

Although theoretical or empirical research makes a case for there being inequalities in ADHD at each stage, no study, to our knowledge, has examined how socioeconomic inequalities manifest along the ‘patient journey’. A better understanding of this will highlight where health or wider services can intervene to provide families with appropriate support, through improving diagnosis and appropriate treatment opportunities for all social groups. The aim of this study is to describe socioeconomic inequalities at each stage of the patient journey for children with ADHD.

## Methods

We describe inequalities at the four ‘stages’ (Figure 1): ADHD symptoms, impact, diagnosis and medication. We use data from two complementary data sets: an ‘administrative cohort’ created from routine records including the Scottish Prescription Information System (all children born in Scotland, 2010–2012, *n* ~ 100,000) and the UK Millennium Cohort Study (MCS, *n* ~ 18,000). The administrative cohort is, to our knowledge, the first of its kind in the UK, providing greater statistical power than cohort surveys, alongside real‐time assessments from health professionals. The MCS is the UK's most recent nationally representative cohort survey. It includes information on parents' perceptions of ADHD symptoms and their impact, and whether the cohort member has been diagnosed with ADHD and received treatment.

### Scottish administrative cohort

We use data from the health and birth records of children born in Scotland between October 2010 and March 2013, followed until April 2018 (median age 6 years), who were included in the Community Health Index (CHI) population register (which provides a health care unique identifier). Records used for the purposes of this analysis were the National Records of Scotland (NRS) Births (which holds information on all registered births), the Prescribing Information System (PIS) (a national database which records all NHS prescriptions that are prescribed, dispensed and reimbursed in the community in Scotland) and Child Health Systems Programme Pre‐School (CHSP‐PS) (which automatically calls and recalls children for scheduled child health review according to the Universal Health visiting pathway (Scottish Government, 2015)).

The use of these anonymised data received NHS Health Scotland Public Benefit and Privacy Panel for Health (PBPP) approval (ref: 1617‐0152); no ethical clearance was required.

#### Measures

Early signs of possible ADHD symptoms are represented by health visitor reports of developmental concerns at age 27–30 months based on any of these three relevant domains: attention, behaviours and social development. We created a binary variable to identify children with a potential issue in at least one of these domains. Sensitivity analyses examined attention concerns only, as these arguably most closely align with ADHD symptoms. Health visitors (or other child health professionals) can choose to use one or more tools to inform these assessments. In this cohort of children, the majority used at least two, with the most common being the Ages and Stages Questionnaire (ASQ) and the Strengths and Difficulties Questionnaire (SDQ).

ADHD medication was defined as any dispensed ADHD prescription for a relevant medication (atomoxetine, dexamfetamine sulphate, guanfacine hydrochloride, lisdexamfetamine dimesylate, methylphenidate hydrochloride—see elsewhere for brands (NHS National Services Scotland, 2019)) until the end‐point of follow‐up (April 2018, median age 6 years). The comparison group was children who had no record of ADHD medication being dispensed.

There is no single measure that can fully reflect a child's socioeconomic circumstances (SECs) (Pearce et al., 2019). In this study, we used the National Statistics Socioeconomic Classification (NS‐SEC) of the mother (occupational status) as it was available and comparable across both data sets. Other benefits of NS‐SEC include that it is an appropriate marker of SECs for children, which is widely used in population research (Clery, Grant, Harron, Bedford, & Woodman, 2022; Pearce et al., 2019); and it usually shows similar health gradients to other SEC measures, such as household income or parental education (e.g. see Lewis, Hope, & Pearce, 2015). NS‐SEC was based on mothers' current or last occupation at birth registration, categorised as Economically Inactive, Routine and Manual, Intermediate, Managerial and Professional. We carried out sensitivity analyses using alternative measures (see ‘Sensitivity analyses’).

#### Analytic sample

A total of 103,596 children had information recorded in the 27–30‐month child health check. Our final analytic sample consisted of those who had data on the relevant development domains and whose mother was not recorded as having left the CHI data set during the study period (an indication of migration out of Scotland) (*n* = 96,718).

### Millennium Cohort Study (MCS)

The Millennium Cohort Study (MCS) is a representative cohort of children born in the UK, 2000–2002. A stratified clustered sampling design was used to oversample children living in Wales, Scotland and Northern Ireland and areas with greater disadvantage and high proportions of ethnic minority groups (in England) (Plewis, 2004). Caregivers were first contacted for interview when the cohort member was age 9 months, with information collected on 18,818 children (72%) of those contacted, (18,296 were singletons, the focus of this paper). Families have been followed up at regular intervals since, with interviews conducted in the home with the main respondent (predominantly the natural mother), partners (where possible and applicable) and, as they have become older, the cohort members themselves. Ethics approval was granted for each MCS survey and no additional ethical approval is required for these analyses.

#### Measures

ADHD symptoms (stage 1) were identified using main respondent‐reported SDQ hyperactivity sub‐scales at 3, 5, 7 years, using the borderline‐abnormal cut‐off to identify caseness, which is related to an increased risk of an ADHD diagnosis (Goodman, 2001).

Impact of these difficulties on day‐to day functioning (stage 2) was identified using SDQ impact questions, also recorded at 3, 5, 7 years, across six domains: home life, classroom, friendships, leisure, the family and upsetting the child. Two or more answers of ‘quite a lot’ or ‘a great deal’ were used to represent impact (and abnormal score) (Kjærandsen, Handegård, Brøndbo, & Halvorsen, 2023). The impact scores have been shown to be a valid indicator of general functional impairment in a paediatric population (Kjærandsen et al., 2023).

ADHD diagnosis (stage 3) is based on the uncorroborated caregiver report of having ever been told by a doctor or other health professional that the cohort child has ADHD at 5, 7, 11 or 14 years. For reports after 5 years, this reflects any current or prior report of ADHD diagnosis (e.g. at 14years, whether the caregiver reported a diagnosis at 5, 7, 11 or 14 years). Caregiver reports have been used in other studies as a measure that is appropriate for the identification of diagnosed ADHD (see, e.g. Russell, Ford, Rosenberg, & Kelly, 2014).

Medication for ADHD (stage 4) was derived from caregiver reports of whether the cohort member, at 14 years, was reported as ‘currently taking any medicines on a regular basis that were prescribed by a doctor or hospital for their ADHD’.

The measure of maternal occupational status used in the administrative cohort was also used in the MCS, at age 9 months.

#### Analytic sample

We analysed data for 8958 cohort members who were present at all relevant sweeps (9 months, 3, 5, 7, 11, and 14 years) and had relevant weights to account for the sample design and attrition up to the 14‐year sweep. Missing data were generally low, with the sample ranging from 8482 to 8884, depending on the outcome.

### Analysis

Prevalences (and *n*) of children displaying ADHD symptoms, impact, diagnosis and medication were estimated, overall and by NS‐SEC, providing a picture of inequalities at each stage in the two data sets. Prevalences and inequalities in the ADHD outcomes were then estimated for those who had reached the previous ADHD ‘stage’. In the administrative data, we estimated the overall prevalence in dispensing of ADHD medication among those who were identified as having relevant developmental concerns at age 27–30 months; due to small cell sizes, it is not possible to present numbers by SECs. In the MCS, we could additionally describe the prevalence and inequalities in: perceived impacts among families who had experienced ADHD symptoms by age 7; ADHD diagnosis among those who had experienced ‘ADHD impacts’ by age 14 and ADHD medication among those who had been diagnosed, also by age 14. Because these analyses require the child to have reached the previous stage of the ADHD patient journey, they are hereafter referred to as ‘conditional analyses’.

Finally, we estimated the relative indices of inequality (RII) and 95% confidence intervals (CIs) at each ADHD stage, overall and conditional on reaching the previous stage. The RII provides a summary measure of relative inequality using mother's occupational status—a ratio between the notionally most and least advantaged individuals in the population. It uses information from each category (weighted to account for category size) across the social gradient, by fitting a line of best fit (Mackenbach & Kunst, 1997).

#### Sensitivity analyses

As a sensitivity analysis, we examined attention concerns in the administrative data (excluding behavioural and social domains). We repeated analyses taking the highest of the mothers' and fathers' (where relevant) occupational status in both data sets and additionally repeated the MCS analyses using quintile of equivalised household income. When reporting the MCS findings, we focus on having ‘ever’ experienced the outcomes by the oldest age listed, since these provide the greatest statistical power. We note meaningful differences at younger ages, however, with cross‐sectional findings for all measurement ages provided in Table S1.

## Results

### Scottish administrative cohort

#### Sample characteristics

The prevalence of ADHD‐like symptoms in the administrative cohort was 9% at age 27–30 months, as reported by the health visitor, in both the total eligible sample and the analytic sample (Table 1). By median age 6, a small minority of all children had been prescribed (and dispensed) ADHD medications (0.15% and 0.14% in the eligible and analytic samples, respectively).

**Table 1:** Characteristics of the administrative cohort

	Eligible sample (103,596)	Analytic sample (96,718)
ADHD‐like symptoms^a^	8.9% (8779)	8.9% (8592)
Missing	4815	N/A
ADHD prescribing		
Any medication dispensed	0.15% (149)	0.14% (136)
Missing	0	N/A
Mother NS‐SEC		
Economically Inactive	20.7% (21,424)	20.5% (19,824)
Routine and manual	26.9% (27,849)	27.0% (26,094)
Intermediate	20.6% (21,360)	20.8% (20,105)
Managerial and professional	31.8% (32,693)	31.7% (30,695)
Missing	0	N/A

^a^ One or more concerns relating to attention, social and behavioural development. NS‐SEC: national statistics socioeconomic classification.

#### Inequalities in ADHD stages

**Figure 2:**
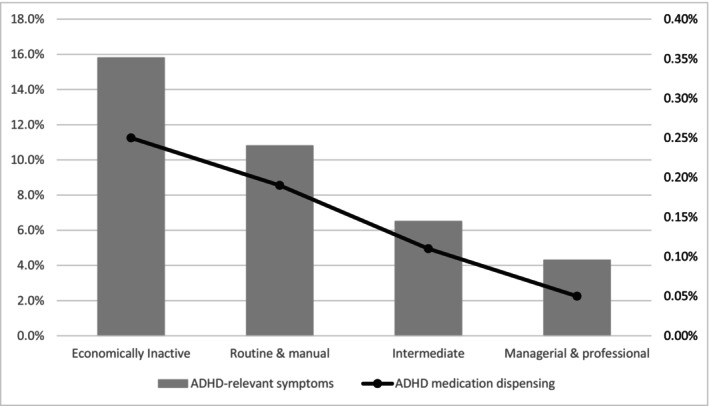
Social gradient in ADHD symptoms (LHS axis) and dispensed medications (RHS axis), administrative cohort.

There was a clear social gradient in ADHD‐relevant symptoms at age 27–30 months, ranging from 4.3% in the most (managerial and professional) to 15.8% in the least advantaged (economically inactive) (Table 2, Section A and Figure 2). Dispensed ADHD medications were also socially patterned, ranging from 0.05% in the most advantaged group to 0.25% in the least advantaged group (Table 2, Section A and Figure 2). The RII for ADHD‐relevant symptoms was 5.9 (95% CI: 5.5, 6.4), increasing to 8.1 (4.2, 15.6) for dispensed ADHD medications.

**Table 2:** Socioeconomic inequalities in the prevalence of ADHD‐relevant symptoms (at 27–30 months) and dispensing of ADHD medications (by median age 6) in the administrative cohort. Overall (Section A) and conditioned on reaching previous stage (Section B)

	Overall	Economically inactive	Routine and manual	Intermediate	Managerial and professional	RII
A. Overall percentages and RII (for each stage)						
1. ADHD‐relevant symptoms^a^ (*n* = 96,718)	8.9% (8592)	15.8% (3130)	10.8% (2827)	6.5% (1313)	4.3% (1322)	5.9 (5.5, 6.4)
4. ADHD medication dispensing (*n* = 96,718)	0.14% (136)	0.25% (49)	0.19% (50)	0.11% (23)	0.05% (14)	8.1 (4.2, 15.6)
B. Percentages and RII among those reaching previous stage						
ADHD medication dispensing to those with symptoms (8592)	0.6% (53)	*Suppressed* ^b^				3.2 (0.99, 10.1)

^a^ One or more concerns relating to attention, social and behavioural development, age 27–30 months; NS‐SEC: national statistics socioeconomic classification.

^b^ % (numbers) suppressed due to small cell sizes.

The conditional analysis (Table 2, section B) showed that 0.6% of children who experienced ADHD‐like symptoms between age 27 and 30 months had a record of ADHD medications being dispensed by age 6 years. While this figure is low, an even smaller proportion of those who had not displayed symptoms in toddlerhood were recorded as having any ADHD medications dispensed by age 6 years (approximately 0.1%, data not shown). Among 27–30 month olds with ADHD‐relevant symptoms, the least advantaged were three times more likely to have ADHD medications dispensed than the most advantaged (RII: 3.2 (0.99, 10.1)).

### UK Millennium Cohort Study

#### Sample characteristics

The prevalences of elevated hyperactivity symptoms were similar in the eligible and analytic samples (Table 3). In both samples, prevalence was highest at age 3 years (eligible sample: 23.9% vs. analytic sample: 24.2%), lowest at 5 years (eligible sample: 17.2% vs. analytic sample: 17.7%) before increasing at 7 years (eligible sample: 20.1% vs. analytic sample: 19.6%). Impact increased with age, from 3 years (eligible sample: 2.1% vs. analytic sample: 2.3%) to 7 years (4.7% in both samples). The prevalence of ADHD diagnosis increased from 5 years (eligible sample: 0.9% vs. analytic sample: 1.0%) to 14 years (eligible sample: 4.4% vs. analytic sample: 3.9%). Prescribed ADHD medication at 14 years was approximately 3% in both samples.

**Table 3:** Characteristics of the UK Millennium Cohort Study

	Eligible sample *n* = 18,296		Analytic sample *n* = 8958	
	Total *n*	% with characteristic	Total *n*	% With characteristic
Hyperactivity symptoms				
Age 3 years	13,855	23.9	8526	24.2
Missing	4441		432	
Age 5 years	13,944	17.2	8711	17.7
Missing	4352		247	
Age 7 years	12,784	20.1	8752	19.6
Missing	5512		206	
ADHD impact				
Age 3 years	13,855	2.1	8526	2.3
Missing	4441		432	
Age 5 years	13,944	2.6	8711	2.5
Missing	4352		247	
Age 7 years	12,784	4.7	8752	4.7
Missing	5512		206	
ADHD diagnosis				
By 5 years (%)	14,390	0.9	8924	1.0
Missing	3906		34	
By 7 years (%)	13,091	1.9	8925	2.0
Missing	5205		33	
By 11 years (%)	12,536	3.3	8910	3.0
Missing	5760		48	
By 14 years (%)	11,032	4.4	8901	3.9
Missing	7264		57	
ADHD prescribing				
By 14 years	11,032	3.4	8902	2.8
Missing	7264		56	
Mother NS‐SEC				
Economically inactive	1932	7.4	664	8.5
Routine and manual	7696	40.0	3212	41.9
Intermediate	3711	22.4	1996	21.7
Managerial and professional	4784	30.2	3036	27.8
Missing	173		50	

NS‐SEC, national statistics socioeconomic classification.

#### Inequalities in ADHD stages

**Figure 3:**
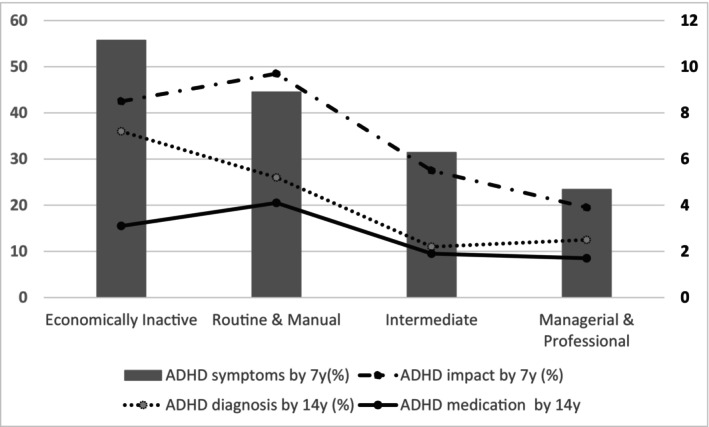
Socioeconomic inequalities in the prevalence of ADHD symptoms (LHS axis), impact, diagnosis and medication (RHS axis), UK Millennium Cohort Study.

There was a social gradient for children having ever experienced ADHD‐relevant symptoms by 7 years, with the prevalence ranging from 23.4% in the most to 55.7% in the least advantaged group (Table 4, Section A and Figure 3).

**Table 4:** Socioeconomic inequalities in prevalence of ever experiencing ADHD‐like symptoms, impact, diagnosis and medication in the UK Millennium Cohort Study, by ages 7 (for stages 1–2) and 14 years (for stages 3–4).^a^ Overall (Section A) and conditioned on reaching previous stage (Section B)

	*n*	Overall %	Economically Inactive %	Routine and Man. %	Intermediate. %	Managerial. and professional. %	RII (95% CIs)
A.Overall percentages and RII (for each stage)							
1. ADHD symptoms by 7 years	8761	36.5	55.7	44.5	31.4	23.4	3.08 (2.68–3.55)
2. ADHD impact by 7 years	8710	7.0	8.5	9.7	5.5	3.9	3.88 (2.68–5.62)
3. ADHD diagnosis by 14 years	8853	4.0	7.2	5.2	2.2	2.5	4.63 (2.47–8.70)
4. ADHD medications by 14 years	8854	2.9	3.1	4.1	1.9	1.7	3.75 (2.21–6.36)
B.Percentages and RII among those reaching previous stage							
1. ADHD symptoms by 3–7 years	8761	36.5	55.7	44.5	31.4	23.4	3.08 (2.68–3.55)
2. ADHD impact by 7 years if symptoms by 7 years	2854	19.5	15.7	22.1	17.6	16.7	1.29 (0.91–1.84)
3. ADHD diagnosis by 14 years, if impact by 7 years	526	26.5	29.0	27.1	20.8	29.3	1.06 (0.50–2.24)
4. ADHD medications by 14 years, if diagnosed by 14 years	282	72.5	44.1	79.0	84.9	67.3	0.84 (0.51–1.39)

ADHD, attention‐deficit hyperactivity disorder; RII, relative indices of inequality.

^a^ Results for all ages provided in Table S1.

As shown visually in Figure 3, there was less evidence of a socioeconomic gradient in impacts from ADHD symptoms, with the Routine and Manual group having a higher prevalence (9.7%) than the Economically Inactive group (8.5%), although prevalence in both groups was considerably higher than in the Managerial and Professional group (3.9%) (see also Table 4, Section A).

Main respondent reports of an ADHD diagnosis (at or before 14 years) were socially graded, with the Economically Inactive group having the highest prevalence (7.2%) and the Intermediate (2.2%) and Managerial and Professional (2.5%) group having the lowest prevalence.

For inequalities in ADHD medication (at 14y), prevalence was lower in the Economically Inactive group (3.1%) than in the Routine and Manual group (4.1%). However, the prevalence of both groups was greater than for the highest social group, Managerial and Professional (1.7%).

The RIIs in Table 4 (section A) show that, overall, inequalities were similar across the four ADHD stages, with potentially some widening at the point of diagnosis, followed by a reduction in inequalities for medication. However, the RII can disguise patterns between groups if the relationships are not linear (e.g. lower medication rates in those in the Economically Inactive group compared to the Routine and Manual group).

The conditional analysis (section B of Table 4) shows prevalence and inequalities at each stage, for those children who had been identified as reaching the previous stage of the ADHD patient journey. Fewer than one in five families (19.5%) whose children were reported as having ADHD‐like symptoms between ages 3 and 7 years felt that there was an impact on daily life. A greater proportion (26.5%) of children who had experienced impact went on to receive a diagnosis by age 14 years and most children (72.5%) who had received a diagnosis received ADHD medications by age 14 years.

Children who were from the least advantaged (economically inactive) households were less likely to be rated by the main respondent as having been impacted by their ADHD‐like symptoms, although children from the most advantaged households (managerial and professional) were also at a lower likelihood of being rated as impacted than the middle groups. There were no clear inequalities between social groups introduced at the point of diagnosis. However, families from the least deprived backgrounds were less likely than any other group to have received medication once diagnosed (44.1% in the Economically Inactive group compared to 84.9% in the Intermediate occupation group). Children in the most advantaged households were less likely to receive medication than any group other than the least advantaged, suggesting a U‐shape relationship between social class and medication following diagnosis. The RIIs (Section B, Table 4) should be interpreted cautiously due to these nonlinear patterns and wide confidence intervals.

### Sensitivity analyses

Findings were similar when we examined inequalities in attention concerns in the administrative data (excluding behavioural and social domains) and when repeating analyses taking the highest of the mothers' and fathers' (where relevant) occupational status (both datasets) and according to household income (MCS only).

## Discussion

### Summary of findings

We found clear social gradients in ADHD‐like symptoms in both data sets. Inequalities were apparent throughout the early years in the MCS, indicating that those seen in Scottish toddlers in the administrative data are likely to have persisted. We also found a steep social gradient in ADHD medications which had been dispensed, at average age 6 in the Scottish administrative cohort. Similarly, in the MCS (at 14 years), children from less advantaged backgrounds were more likely to have received medication.

Additional information on impact of symptoms and diagnosis was available in the MCS and suggests that patterns of inequality along the patient journey from symptoms to treatment are complex. In general, children from economically inactive and routine and manual groups were more likely to experience all ADHD stages than those from intermediate and managerial professional groups. However, there was not always a typical (linear) social gradient. Furthermore, the conditional analysis showed that children from less advantaged backgrounds who were experiencing ADHD‐relevant symptoms were *less* likely to have reported by the main caregiver that those symptoms were impacting on daily life, when compared to children from more advantaged backgrounds. Additionally, children from the economically inactive group were the least likely to receive medication, following diagnosis, potentially indicating that the Inverse Care Law applied (Hart, 1971). Those in the most advantaged group (managerial and professional) also had relatively low levels of medication, possibly suggesting that parents from this advantaged group are more likely to explore preferred, nonpharmaceutical alternatives, although we could not test this with the data available.

### Comparison with other findings

Inequalities in ADHD symptoms, diagnosis and treatment have been widely documented. A model of ADHD help‐seeking behaviour has previously been used to consider ethnic inequalities in ADHD, proposing that these may manifest through differences in problem recognition, decision to seek help, service selection and service utilisation (Eiraldi et al., 2006). The stages we have considered (symptoms, impact, diagnosis and treatment) are different from those reported in previous research but not incompatible. To our knowledge, only one study has examined how socioeconomic inequalities might arise at multiple stages. Russell et al. (2014) sought to investigate the role of clinical labelling bias in explaining the higher risk of ADHD diagnosis in children from less advantaged families in the Millennium Cohort Study at age 7 years. They did this by adjusting for ADHD symptoms and impacts (using parent and teacher SDQ reports) at the same age. Because inequalities in ADHD diagnosis were removed after adjustment, the authors concluded there was no clinical labelling bias. We have extended this work by investigating how inequalities emerge at each of the four proposed stages through to adolescence in the MCS, while also replicating some elements of the analyses using population‐wide administrative data. Our findings point towards a complex picture. For example, the conditional analysis across the ADHD patient journey indicated that those in the most advantaged group were among the least likely to experience ADHD symptoms and any impact of those symptoms. However, once impact had been established, they were among the most likely to receive a diagnosis, although they had a relatively low likelihood of receiving medications after diagnosis.

A 2015 systematic review identified four themes with regards to the barriers and facilitators to ADHD care: ‘wider determinants’ (which included social networks and urban residence, as well as ethnicity and markers of socioeconomic position), identification of need, entry and continuity of care, and interventions to improve access (Wright et al., 2015). While they did not explicitly examine the interaction between the ‘wider determinants’ and the other three types of barriers, their findings may shed light on some of the patterns we have observed. For example, the lower likelihood of perceived impact in less advantaged families may be due to differences in parental expectations, while the lower likelihood of receiving medication after diagnosis may be explained by some families being less well equipped to navigate the health care system than others, as illustrated by the Inverse Care Law (Hart, 1971). Children with diagnosed ADHD from the most advantaged backgrounds also had a relatively low likelihood of receiving medications; this may be because their parents are more able to successfully explore and identify nonmedical treatment options if these are preferred over pharmacological treatment. Unfortunately, treatments such as cognitive behavioural therapy or changes to family routines were not reported in the MCS or identifiable in the administrative data available to us. Future research should seek to examine if family socioeconomic circumstances are associated with other treatments.

### Strengths and limitations

We have described inequalities at several, measurable stages of the ADHD ‘patient journey’. We may have overlooked some important stages in the ADHD process—for example, it was not possible to explore whether medication was taken once it had been prescribed. Additionally, we could not explore the mechanisms through which these inequalities arose or the role of teachers or schools in highlighting potential symptoms and supporting families on the patient journey.

Despite using administrative data and a large UK cohort survey, this study was relatively underpowered to investigate the ADHD patient journey, with relatively large confidence intervals around some inequalities estimates. We relied on the time series of main respondent (generally a parent) reports of ADHD outcomes in the MCS, which may have introduced response bias. The ADHD prescribing data only refer to those coming via the NHS and do not include prescriptions from private healthcare providers. Thus, we may have underestimated prescriptions in more advantaged groups, although self‐reports of medication in the MCS will not be susceptible to this bias. We cannot directly compare the findings from the MCS and the administrative data, which had arguably more ‘objective’ measures, due to differences in the timing of measurement, instruments used, and the fact that the two cohorts were born approximately 10 years apart. Nevertheless, social patterning was apparent in both data sets.

The administrative data provided a shorter follow‐up period, and ADHD symptoms could only be indirectly assessed at an early developmental age (27–30 months), when ADHD symptoms may be hard to detect. Furthermore, we used a holistic measure of child developmental concerns, covering social, attention and behavioural domains. While sensitivity analyses limited to the attention domain only showed similar findings, it is likely that many of these children were experiencing neurodevelopmental problems not related to ADHD. Health visitors could use a range of tools to inform their assessments, and in this particular cohort, the most commonly used tools were the Ages and Stages Questionnaire and the SDQ. While this may create some variability in the outcome, it is unlikely to have caused bias in the inequalities observed.

The marker of socioeconomic circumstances (SECs) adopted (mother occupational status) has limitations. For example, the ‘Economically Inactive’ is a heterogeneous group, including students as well as the long‐term unemployed. Nevertheless, occupational status was chosen because it was available in both data sets and is considered to be a better marker of family SECs than area‐level measures of deprivation which describe neighbourhoods rather than families and which will be confounded by variations in health service provision and quality in different areas. Similar social gradients in ADHD to those shown for occupational status in our analyses have been observed for other SEC measures, such as income and parental education (Russell et al., 2016), suggesting that the results we report are not an artefact of the particular measure of SEC adopted. This was confirmed by sensitivity analyses in the MCS using household income. We also repeated analyses taking the highest NS‐SEC of both parents (where relevant) and findings were unchanged.

### Implications for policy, practice and further research

Future studies should explore the interval between symptoms, diagnosis and medication, as this could indicate barriers to treatment within the health system. Qualitative research would provide a better understanding of the barriers and supports at different stages of the ADHD patient journey, and thus the mechanisms through which the inequalities we have observed arise. Nevertheless, our finding that children from the least advantaged families are less likely to report day‐to‐day impact of ADHD symptoms, once they have been experienced, and are also less likely to be prescribed medication, postdiagnosis, suggests possible points in the system where professionals may be able to intervene to ensure that children are receiving sufficient and appropriate support. Due to the potential of stigma and other psychosocial impacts of receiving an ADHD diagnosis, recognition and confirmation of ADHD symptoms may only be beneficial to the child if acceptable and effective treatments are available to support them (Wright et al., 2015).

## Funding

This work was supported by an award to AP from Wellcome (205412/Z/16/Z). AP, SVK, AL and RD are also supported by the Medical Research Council (MC_UU_00022/2) and the Scottish Government Chief Scientist Office (SPHSU17).

## Conflict of interest

The authors have declared that they have no competing or potential conflicts of interest.

## Ethical information

No ethical approval was required for the secondary data analyses. The use of these anonymised data received NHS Health Scotland Public Benefit and Privacy Panel for Health (PBPP) approval (ref. 1617‐0152). Ethics approval was granted for each individual Millennium Cohort Study survey.

## Supporting information


**Table S1.** Percentages and RIIs for ADHD outcomes, according to maternal occupational status which includes cohort members in all (MCS1‐6) sweeps (*n* = 8958).
