# Supplementary material for: Childhood attention‐deficit hyperactivity disorder: socioeconomic inequalities in symptoms, impact, diagnosis and medication
Source: Child Adolesc Ment Health. 2024 Mar 18;29(2):126–35. doi: 10.1111/camh.12707 (PMC11963976; doi:10.1111/camh.12707)
Supplement: Supplementary file 1 — Table S1. Percentages and RIIs for ADHD outcomes, according to maternal occupational status which includes cohort members in all (MCS1‐6) sweeps (n = 8958). [file CAMH-29-126-s001.docx]

*Childhood attention-deficit hyperactivity disorder (ADHD): Socio-economic inequalities in symptoms, impact, diagnosis and medication*

**Online only supplementary materials**

Table A1: Percentages and RIIs for ADHD outcomes, according to maternal occupational status. Includes cohort members in all [MCS1-6] sweeps (*n*= 8,958).

|  | *n* | Overall % | Economically Inactive % | Routine & Manual % | Intermediate. % | Managerial & professional. % | RII (95% CIs) |
| --- | --- | --- | --- | --- | --- | --- | --- |
| *ADHD-like symptoms* | | | | | | | |
| 3y (%) | 8482 | 24.2 | 35.7 | 31.2 | 20.4 | 14.4 | 3.58 (2.94-4.37) |
| 5y (%) | 8664 | 17.7 | 31.4 | 22.7 | 13.5 | 10.2 | 4.38 (3.47-5.55) |
| 7y (%) | 8707 | 19.5 | 31.1 | 24.1 | 17.1 | 11.7 | 3.47 (2.79-4.31) |
| *ADHD impact* | | | | | | | |
| 3y (%) | 8482 | 2.3 | 2.6 | 3.7 | 1.4 | 1.1 | 6.46 (3.09-13.49) |
| 5y (%) | 8664 | 2.5 | 3.1 | 3.1 | 2.1 | 1.8 | 2.47 (1.38-4.42) |
| 7y (%) | 8707 | 4.7 | 4.3 | 6.3 | 4.4 | 2.7 | 2.95 (1.92-4.52) |
| *ADHD diagnosis* | | | | | | | |
| By 5y (%) | 8874 | 1.1 | 3.2 | 1.2 | 0.3 | 0.8 | 7.71 (1.13-52.68) |
| By 7y (%) | 8875 | 2.0 | 5.2 | 2.3 | 1.1 | 1.3 | 5.91 (1.82-19.16) |
| By 11y (%) | 8860 | 3.0 | 6.4 | 3.9 | 1.7 | 1.8 | 5.61 (2.52-12.47) |
| By 14y (%) | 8853 | 4.0 | 7.2 | 5.2 | 2.2 | 2.5 | 4.63 (2.47-8.70) |
| *ADHD medications* | | | | | | | |
| At 14y (%) | 8854 | 2.9 | 3.1 | 4.1 | 1.9 | 1.7 | 3.75 (2.21-6.36) |

Sample varies between analyses due to missing values for analytic variables at each sweep. All analyses weighted using MCS6 survey/response weight. MCS: Millennium Cohort Study. RII: relative index of inequality. CI: 95% confidence interval. ADHD: attention-deficit hyperactivity disorder. Y: years.
